# BioTM buzz

**DOI:** 10.1002/btm2.10057

**Published:** 2017-03-27

**Authors:** Aaron Anselmo

This special issue of *Bioengineering & Translational Medicine* focuses on the development of bioengineered therapeutics; specifically, design and discovery approaches, delivery platforms, and synthesis and manufacturing technologies. These three areas are essential in ensuring the successful clinical translation of new biotechnologies. As such, this first BioTM Buzz will highlight an article in each of these important areas—two articles from this special issue and another from the recent literature.

## Special issue

### Keep up the cross‐talk

Design and discovery approaches are essential in isolating and selecting lead candidates that hold promise as robust therapeutics. In this issue of *Bioengineering & Translational Medicine*, engineers from the Jennifer Cochran lab at Stanford University describe an approach to engineer vascular endothelial growth factor (VEGF) for increased binding affinity to vascular endothelial growth factor receptor 2 (VEGFR2), which inhibits VEGF‐mediated signaling and thereby reduces angiogenesis. The authors had previously reported the design of VEGF‐proteins that enhance VEGF's natural antiangiogenesis properties. In the current study, high‐throughput yeast surface display was used to generate highly‐potent VEGF‐proteins that exhibit enhanced binding affinity to VEGFR2. Enhanced binding of VEGF‐variants was confirmed in mammalian cells, which also led to inhibition of VEGF‐mediated cell signaling and angiogenic processes in mice. The versatile approach will find utility in the design of new therapeutics to optimize ligand–receptor interactions or to improve binding interactions for already‐approved or currently investigated protein‐based therapeutics in the clinic.

DOI: 10.1002/btm2.10051

### In tandem interplay

Biological barriers and other physiological challenges often limit the ability of therapeutics to interact optimally, or at all, with diseased tissues. Chemical engineer Julie Champion and coworkers from the Georgia Institute of Technology describe methods to systematically investigate how the physical properties of particle‐based drug delivery systems influence their fate and function. The particle‐based drug delivery systems that have been clinically approved or investigated each possess unique physicochemical properties; as such, defining particle properties that exhibit enhanced biological performance in humans is a significant challenge that if addressed could aid in the design of future delivery systems. In the current study, a layer‐by‐layer templating approach that enabled control over particle size, shape, and stiffness, while maintaining identical surface chemistry, was used to investigate the individual and combined contributions these modifications have on particle internalization by macrophages. Complex relationships between size, shape, and stiffness were revealed—for example, spherical particles of increased size exhibited reduced internalization by macrophages, while modifications to stiffness exhibited no differences in internalization. The implications described in this paper highlight how current efforts in particle‐delivery must consider all physicochemical properties of particles in tandem, since the interplay between these properties and their biological performance is a complex relationship.

DOI: 10.1002/btm2.10052

## Recent literature

### Transient and reversible

Synthesis and manufacturing technologies that enable the scaled‐synthesis of both therapeutics and delivery platforms are a prerequisite for clinical impact. Often times, complex approaches at the lab‐scale face difficulties in translation towards industrial and commercial processes. An approach developed by Eric Appel at Stanford University and collaborators including Robert Langer at the Massachusetts Institute of Technology enables the scalable manufacturing of hydrogel materials with complex viscoelastic properties. Hydrogels are widely used as a vehicle to enable the controlled release of drugs in a variety of delivery applications. However, cost issues and scalability related to consistency of the synthesized hydrogels have limited their clinical translation. In this study, hydrogels are formed by mixing polymers and hard nanoparticles (the authors report the linear scaled‐synthesis of up to 15 L using this self‐assembly process); since polymer adsorption to nanoparticles is transient and reversible the resulting hydrogels exhibit both shear‐thinning and self‐healing properties. These shear‐thinning hydrogels have great potential for biotechnology applications since they can be readily injected and capable of flowing under high stress, but then form a gel when relaxed. The approach described represents a strategy that could be readily translated to enable the large‐scale synthesis of hydrogels for biomedical applications, such as combination therapies relying on the delivery of drugs of distinct physical properties.

*Yu et al., Proc Natl Acad Sci USA. 2016; doi:* 10.1073/pnas.1618156113

